# The Effects of Traditional Chinese Exercise in Treating Knee Osteoarthritis: A Systematic Review and Meta-Analysis

**DOI:** 10.1371/journal.pone.0170237

**Published:** 2017-01-25

**Authors:** Yingjie Zhang, Lulu Huang, Youxin Su, Zhengxuan Zhan, Yanan Li, Xingquan Lai

**Affiliations:** 1 Department of Rehabilitation Medicine College, Fujian University of Traditional Chinese Medicine, No.1 Qiuyang Road, Shangjie, Minhou, Fuzhou, Fujian, People’s Republic of China; 2 Department of Traumatology and Orthopedics College, Fujian University of Traditional Chinese Medicine, No.1 Qiuyang Road, Shangjie, Minhou, Fuzhou, Fujian, People’s Republic of China; University of Umeå, SWEDEN

## Abstract

**Background and Aims:**

Traditional Chinese exercise (TCE) includes a variety of exercise, which is being accepted by more and more people in the treatment of knee osteoarthritis (OA) from different countries. With the attendant, many clinical reports focus on it. Our meta-analysis aimed to systematically assess the effects of traditional Chinese exercise on pain, stiffness, physical function, quality of life, mental health and adverse events in people with knee osteoarthritis.

**Methods:**

PubMed, Embase, Cochrane Central Register of Controlled Trials (CENTRAL), the Web of Science, and Chinese Biomedical Literature Database (CBM) were searched from the time of their inception through April 2016 and risk of bias was independently assessed by two authors. Outcome measures included pain, physical functional, joint stiffness, quality of life, mental health and safety. For pooled outcomes, standardized mean differences (SMD) and 95% confidence intervals (CI) were calculated.

**Results:**

Eight randomized controlled trials with a sample size of 375 cases met the criteria to be included in the study indicating that high quality literature is lacking in this field. Results of the meta-analysis showed that short-term TCE could relieve pain (SMD: -0.77;95% CI: -1.13 to -0.41; P<0.0001), improve physical function (SMD -0.75; 95% CI: -0.98 to -0.52; P<0.00001), and alleviate stiffness (SMD: -0.56; 95%: CI -0.96 to -0.16; P<0.006), but had no significant effect on quality of life (SMD: 0.57; 95% CI: 0.17 to 0.97; P = 0.005), and mental health (SMD 4.12; 95% CI: -0.50 to 8.73; P = 0.08). Moreover, TCE was not associated with serious adverse events.

**Conclusions:**

Our systematic review revealed that short-term TCE was potentially beneficial in terms of reducing pain, improving physical function and alleviating stiffness. These results may suggest that TCE could prove useful as an adjuvant treatment for patients with knee OA. Further studies are urgently needed to confirm these results.

## Introduction

Knee osteoarthritis (OA) is becoming increasingly prevalent among older adults [1] and is the major cause of chronic pain and disability worldwide [2]. Knee OA often causes pain and stiffness in the affected joint, often leading to a sharp decrease in knee strength and a slowing of gait speed that is beyond what is normally expected due to advancing age [3]. The occurrence of knee OA is often associated with destruction of the articular cartilage in addition to underlying bony changes at the joint margins. And the main symptoms are significant pain, functional limitations which seriously affects quality of life [4,5] and can even cause mental and physical distress [6].

As advanced aging in the population is a continuous process, the treatment of knee OA is in constant demand [7]. Non-operative management approaches are often utilized for the symptomatic treatment of knee OA. Such approaches include physiotherapy, drug therapy [8–12], and exercise regimens, such as aquatic exercise [13], strength training [14], aerobic exercise [15], etc.

Therapeutic exercise has been investigated as a potential knee OA management method [16,17] and is recommended as a non-operative and non-pharmacologic treatment for knee OA in numerous guidelines [18–20].

Traditional Chinese exercise (TCE) is one type of therapeutic exercise, and its practice is increasing globally, with training such as Tai Chi, Qigong and Baduanjin substantially benefiting human health for over 2000 years [21–25]. Compared with other knee OA exercise therapy, such as resistance exercise which is also proved effective on pain relief, stiffness alleviation, and physical function improvement [26], there are studies that show TCEs do not only has a good effect on knee OA, but also have an overall adjustment of health which are mind–body exercises that enable individuals to focus on coordinating various postures with breathing patterns and meditation [27]. TCEs include Tai Chi, Baduanjin, Yijinjing, and Wuqinxi, and are defined as a low-level aerobic exercise that can improve limb range of motion, strength, and general health. However, they also have their own characteristics. For example, Tai Chi exercise featured gentle, graceful, smooth, coordinated and flowing movements of different body parts, stressing constantly shifting of body weight between two legs with both knees slightly flexed all the time [28]. According to the different moves, Tai Chi can be divided into Chen-style Tai Chi and Yang-style Tai Chi, which has gained increasing popularity as a treatment for older adults with knee OA, and it has been shown to significantly enhance strength, balance, flexibility, self-efficacy, and relieve pain, depression, and anxiety in diverse patient populations with chronic conditions [29]. Moreover, the benefits of Tai Chi have been reported in large-scale RCTs and reviews [29,30]. Compared with Tai Chi, the Baduanjin can be learned more easily and is less cognitively and physically demanding [31], which is defined as a low-level aerobic exercise and mainly consists of eight basic actions which can be beneficial to the limbs’ range of motion, strength, and general health [32].

To our knowledge, there have been three recent reviews [33–35] summarizing the effects of Tai Chi on knee OA. Moreover, several studies [32,36–39] have reported the effective use of TCEs in relieving pain, improving physical function, alleviating stiffness, and reducing the progression of osteoarthritis through a series of dance-like movements that combine various postures and forms. Further, movement therapy often combines the slow, smooth, and specific postures with meditative aspects in order to potentially reduce stress and increase psychological well-being.

Although TCEs have been widely performed for the prevention and treatment of knee OA, no consensus has been reached about the benefits of these exercises in the maintenance of physical function and quality of life of patients with knee OA or for the prevention of depression among this population. We are also unaware of any systematic reviews that have assessed the short- and long-term effects of TCE on physical function, quality of life, and mental health among patients with knee OA.

Therefore, this study applied the principle of evidence-based medicine of TCE to perform a systematic review/meta-analysis on the effects on pain, physical function, quality of life, mental health and adverse events of treatment regimens for knee OA, thereby providing guidance and references for further application of this form of therapeutic exercise.

## Materials and methods

### Database and search strategies

The following electronic databases were searched from the time of their inception through April 2016: PubMed (1959–2016), Embase (1980–2016), Cochrane Central Register of Controlled Trials (CENTRAL, 1996–2016), the Web of Science (1900–2016), and Chinese Biomedical Literature Database (CBM) (1978–2016). The journal languages were restricted to Chinese and English. The literature search was constructed around terms for TCE and knee OA, which were adapted for each database as necessary. For example, the search strategy (S1 File) for PubMed was as follows: ("osteoarthritis, knee"[MeSH Terms] OR ("osteoarthritis"[All Fields] AND "knee"[All Fields]) OR "knee osteoarthritis"[All Fields] OR ("osteoarthritis"[All Fields] AND "knee"[All Fields]) OR "osteoarthritis, knee"[All Fields]) AND ("TCE"[All Fields] OR "baduanjin"[All Fields] OR ("yi"[All Fields] AND "jinjing"[All Fields]) OR "wuqinxi"[All Fields] OR "Tai Chi"[All Fields] OR "T'ai-chi"[All Fields] OR "Tai Ji"[All Fields] OR "Taiji"[All Fields] OR "Taiji quan"[All Fields]).

Reference lists of identified original or review articles were searched manually for additional relevant articles.

### Study selection

#### Types of studies

All reports included in this study were clinical randomized controlled trials (RCTs).

#### Types of participants

According to the diagnostic criteria created by the American College of Rheumatology [40,41], all subjects included in the study had been diagnosed with unilateral or bilateral knee OA. Subject ages ranged from 50 and 70 years, and sex, disease duration, and severity were unrestricted. Subjects with knee trauma or surgery, and/or history of rheumatoid arthritis were excluded. Subjects with cognitive impairment were also excluded.

#### Types of interventions

Trials were divided into TCE and control groups based on the intervention methods, with the TCE group receiving TCE alone and the control group receiving Wellness education or no treatment which were eligible.

#### Types of outcome measures

Studies were eligible if they assessed at least one of the following outcome measures: pain, physical function or joint stiffness. If available, data on quality of life, mental health and safety served as secondary outcome measures.

Pain, physical function, and stiffness were measured on a visual analog scale, a numerical rating scale, or on the Western Ontario and McMaster Universities Osteoarthritis Index (WOMAC) scale, which is used worldwide and employed in most knee OA subjects [42]. Patient quality of life and mental health was assessed using the SF-36. Safety was defined as the number of adverse events that occurred during the study or the number of dropouts due to health problems.

### Data extraction

Two reviewers independently screened titles and abstracts of retrieved studies to exclude obviously irrelevant studies. Full texts of the potential studies were then reviewed to determine eligible trials. Articles were considered relevant based on the selection criteria. Then, data was extracted on study characteristics such as participants, interventions, duration, frequency, outcome assessment, outcome measures, and results. During this process, all disagreements between reviewers were resolved by discussion.

### Quality assessment

Study quality was assessed independently by two reviewers using Cochrane Collaboration’s tool to analyze the risk of bias [43]. Recommended domains were selection, performance, detection, attrition, and reporting biases. If study data were inconclusive, the trial authors contacted for further details. Disagreements between reviewers were resolved through discussion.

### Measures of treatment effect

If at least two studies of comparable TCE protocols and outcome measures existed, a meta-analysis was conducted using Review Manager Software (version 5.3, updated 2014 Cochrane Collaboration). A standardized mean difference (SMD) with 95% confidence intervals (CI) was calculated and all P values were two-sided. Overall effect sizes were judged based on Cohen’s categories: effect sizes of 0.2–0.5 was considered a small effect, 0.5–0.8 a moderate effect, and > 0.8 a large effect [44].

### Assessment of heterogeneity

Statistical heterogeneity between studies was tested by calculating Higgins I^2^ values or using the chi-square test. I^2^>25%, I^2^>50%, and I^2^>75% were defined to indicate moderate, substantial, and considerable heterogeneity, respectively [45]. When the P-value of this test was < 0.1, an I^2^ test was carried out. If the I^2^ test showed a value > 50%, indicating substantial heterogeneity and a random effects model was carried out. Otherwise, a fixed effects model was carried out [46]. Sensitivity analysis was performed by excluding the inclusion of the study, in order to determine whether the results of meta-analysis was stable and find the source of heterogeneity [47].

## Results

### Study selection and characteristics

**Fig 1:**
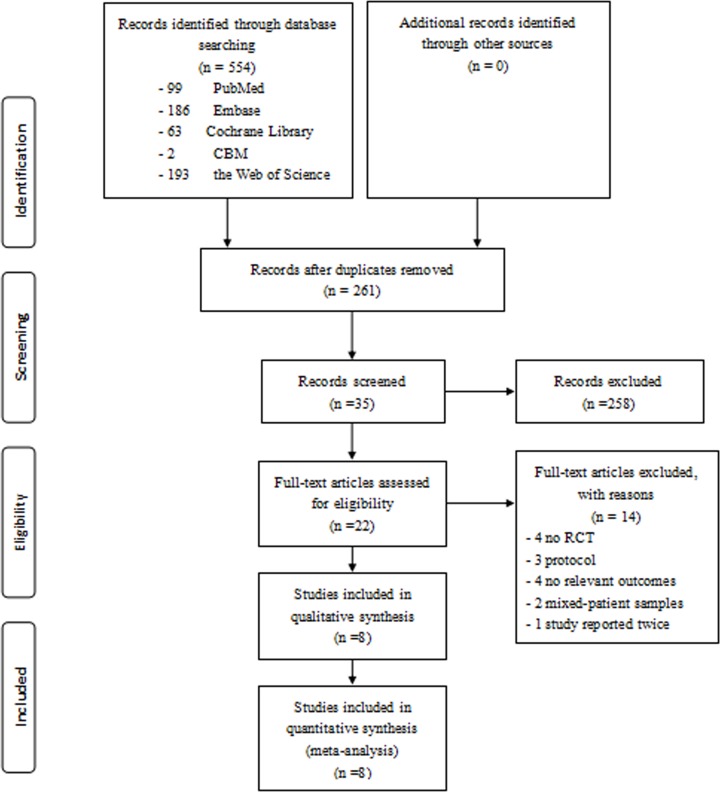
Flowchart of the results of the literature search.

A total of 554 papers were identified from the database searches, 261 of which were duplicates (Fig 1). Twenty-two full-text articles were assessed for eligibility [14,32,38,39,28,48–63] of which, two full-text articles were excluded because they involved mixed patient samples [50,56], four were not RCTs [39,53,60,61], four had no relevant outcomes [14,55,62,63], one was reported twice [49], and three were only a protocol [38,58,59]. Eight studies involving 375 patients were included in our qualitative and quantitative analyses [32,28,48,49,51,52,54,57], and characteristics of the included studies, including samples, interventions, durations, frequencies, outcome assessment, outcome measures, and results, are presented in Table 1.

**Table 1:** Characteristics of The Included Studies.

Author, year	Sample size I/C	Mean age (year) ± SD I/C	Intervention methods (/d) I/C	Duration, frequency I/C	Outcome assessment	Outcome measures 1) Pain 2)Physical function 3) Stiffness 4) Quality of life 5) Mental health 6) Safety	Results a) Short-term follow-up b) Long-term follow-up 1) Pain 2) Physical function 3) Stiffness 4) Quality of life 5) Mental health 6) Safety
**Fransen, 2007 [49]**	**56/41**	**70.8 ± 6.3 69.6 ± 6.1**	**Tai Chi vs Wait-list (no treatment)**	**12 weeks Attend classes for 1 hour, twice a week**	**Pretest–posttest (12 weeks), and follow-up (24 weeks)**	**1) WOMAC** **2) WOMAC** **3) NA** **4) NA** **5) NA** **6)Adverse events**	**a)1)Tai Chi> Wait-list** **2) Tai Chi> Wait-list** **3) NA** **4) NA** **5) NA** **6) one withdrew from Tai Chi due to exacerbation of low back painb) 1) NS** **2) NS** **3) NS** **4) NA** **5) No serious adverse events related to Tai Chi**
**Brismee, 2007 [28]**	**22/19**	**70.8 ± 9.8 68.8 ± 8.9**	**Yang-style Tai Chi vs Attention control**	**18 Weeks Weeks 1–6: Yang-style Tai Chi 3×40 min a week Weeks 7–12: video based home Tai Chi exercise Weeks 13–18: no exercisevs Weeks 1–6: Health lectures and discussions Weeks 7–18: no Lectures**	**Pretest–posttest (12 weeks), and follow-up (18 weeks)**	**1) VAS** **2) WOMAC** **3) WOMAC** **4) NA** **5) NA** **6) Adverse events**	**a) 1) Tai Chi> Attention control** **2) Tai Chi> Attention control** **3) NS** **4) NA** **5) NA** **6) No serious adverse eventsrelated to Tai Chib) 1) NS** **2) NS** **3) NS** **4) NA** **5) NA** **6) No serious adverse eventsrelated to Tai Chi**
**Wortley, 2013 [48]**	**12/6**	**68.1 ± 5.3 70.5 ± 5.0**	**Tai Chi vs No treatment**	**10 weeks 1 h group training session twice a week**	**Pretest–posttest (10 weeks)**	**1) WOMAC** **2) WOMAC** **3) WOMAC** **4) NA** **5) NA** **6) NA**	**a) 1) NS** **2) NS** **3) NS** **4) NA** **5) NA** **6) NA**
**An, 2008 [32]**	**14/14**	**65.4 ± 8.2 64.6 ± 6.7**	**Baduanjin vs No treatment**	**8 weeks 30 min classes five times a week**	**Pretest–posttest (8 weeks)**	**1) WOMAC** **2) WOMAC** **3) WOMAC** **4) SF-36** **5) SF-36** **6) Adverse events**	**a)1) Baduanjin> Attention control** **2) Baduanjin> Attention control** **3) Baduanjin> Attention control** **4) NS** **5) NS** **6) No adverse events were reported**
**Lee, 2009 [51]**	**29/15**	**70.2 ± 4.8 66.9 ± 6.0**	**Tai Chi Qigong vs Wait-list (no treatment)**	**8 weeks 2×60 min a week**	**Pretest–posttest (8 weeks)**	**1) WOMAC** **2) WOMAC** **3) WOMAC** **4) SF-36** **5)SF-36** **6) NA**	**a) 1) Tai Chi> Wait-list** **2) NS** **3) NS** **4) Physical component score: TaiChi> Wait-list** **5)Mental component score: TaiChi> Wait-list** **6) NA**
**Wang, 2009 [57]**	**20/20**	**63.0 ± 8.1 68.0 ± 7.0**	**Yang-style Tai Chi vs Attention control (wellness education and stretching, dietary advice)**	**48 weeks weeks 1–12: Yang-style Tai Chi 2×60 min a week + 20 min home practice a day Weeks 13–48: home practice**	**Pretest–posttest (12 weeks), and follow-up (24 weeks)**	**1) WOMAC** **2) WOMAC** **3) WOMAC** **4) SF-36** **5) SF-36** **6) Adverse events**	**a) 1) Tai Chi> Attention control** **2) Tai Chi> Attention control** **3) NS** **4) Physical component score: TaiChi> Attention control** **5) Mental component score: TaiChi> Attention controlb) 1) Tai Chi> Attention control** **2) NS** **3) NS** **4) NS** **5) NS** **6) No serious adverse eventsrelated to Tai Chi**
**Song, 2003 [54]**	**22/21**	**64.8 ± 6.0 62.5 ± 5.6**	**Sun-style Tai Chi vs Wait-list (continuing standard care)**	**12 weeks Weeks 1–2: 3×12 Tai Chi movements a week Weeks 3–12: 1×12 Tai Chi Movements a week + video based Tai Chi exercise at home, at least 3× 20 min a week**	**Pretest–posttest (12 weeks)**	**1) WOMAC** **2) WOMAC** **3) WOMAC** **4) NA** **5) NA** **6) NA**	**a) 1) Tai Chi> Wait-list** **2) Tai Chi> Wait-list** **3) Tai Chi> Wait-list** **4) NA** **5) NA** **6) NA**
**Ni, 2010 [52]**	**18/17**	**62.89 ± 2.79 63.47 ± 2.85**	**Yang-style Tai Chi vs Attention control (wellness education and stretching)**	**24 weeks Weeks 1–8: 2 days a week Weeks 2–16: 3 days a week Weeks 16–24: 4 days a week vs 45-minute wellness education and stretching sessions, once a week**	**Pretest–posttest (24 weeks)**	**1) WOMAC** **2) WOMAC** **3) WOMAC** **4) NA** **5) NA** **6) Adverse events**	**a) 1) Tai Chi> Attention control** **2) Tai Chi> Attention control** **3) Tai Chi> Attention control** **4) NA** **5) NA** **6) No serious adverse eventsrelated to Tai Chi**

Abbreviations: I/C: intervention /control group; WOMAC: Western Ontario and McMaster Universities Osteoarthritis Index; SF-36, Short Form Health Survey; NA, Not assessed; NS, Not significant; SF-36, Short Form Health Survey; VAS, Visual Analog Scale.

Trials originated from the United States [28,48,57], Australia [49], Korea [51,54], and China [32,52]. Patients were recruited from outpatient clinics [54], local community centers [32,51], or through advertisements in local newspapers, flyers, and other media [28,48,49,52,57]. Patients were in their 50s–70s, and most were female. Among TCEs employed, Tai Chi [28,48,49,51,52,54,57] was used the most for treating knee osteoarthritis, and Baduanjin [32] was also used to treat knee OA. TCE intervention duration ranged from 8 to 24 weeks, of which 12 weeks accounted for the majority. And the most commonly utilized frequency was delivered in great difference, of which twice a week was the most.

The majority of the included studies used the WOMAC scale to measure pain, physical function, and stiffness, while the SF-36 was most commonly used to measure quality of life and mental health. One study [28] used the VAS to measure pain after short-term TC, and five studies reported adverse events of short-term TCE [9,11,13,16,22]. The long-term effects of TCE were only reported in three studies.

### Risk of bias in included studies

**Fig 2:**
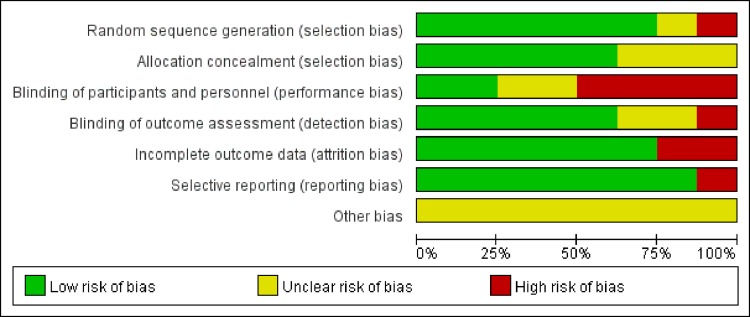
Risk of bias graph: review authors' judgments about each bias item, presented as percentages.

**Fig 3:**
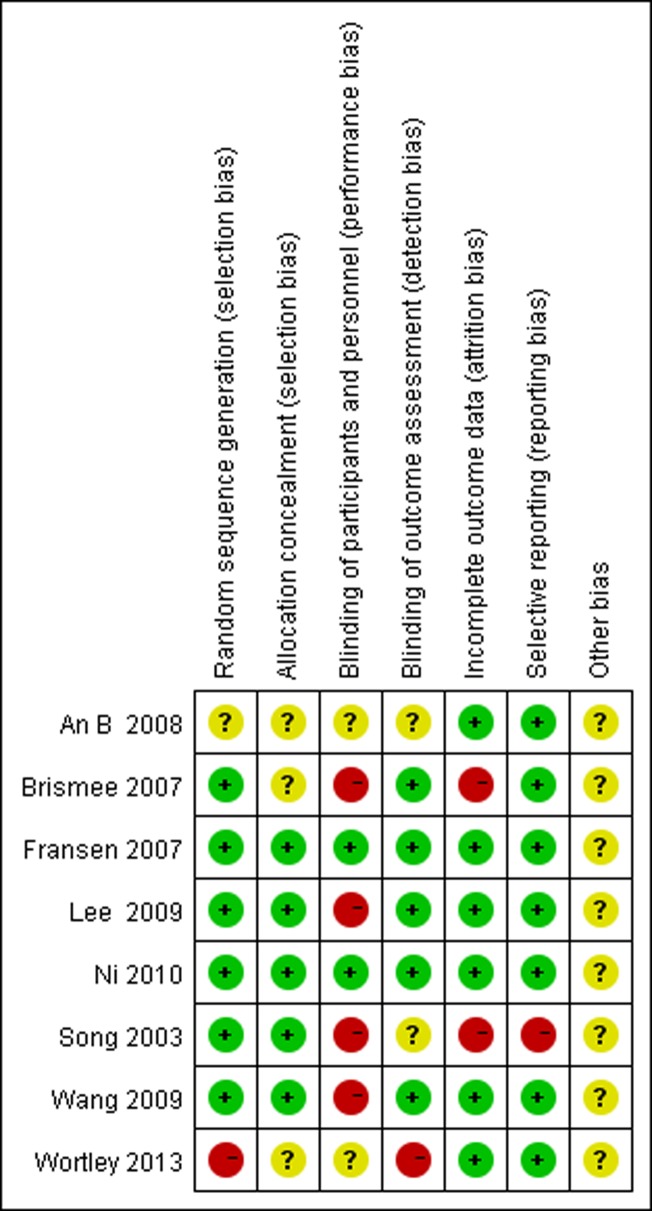
Risk of bias summary: review authors' judgments about bias items for each included study.

According to the Cochrane Collaboration, none of the studies received the maximum quality score (Figs 2 and 3). In six of the included studies, random sequences were generated via a random table or computer. One study generated sequences pseudo-randomly [48] and the other did not mention it [32]. Lack of double blinding was the most common source of likely methodological bias. Only two of the eight included studies reported blinding of both participants and outcome assessors [49,52]. However, in our assessments of blinding, five of the included studies presented low risk [28,49,51,52,57]. For the item incomplete outcome data two studies presented high risk [28,54] and six studies exhibited low risk [32,48,49,51,52,57]. More information is urgently needed to confirm other biases.

### Effect of interventions

**Fig 4:**
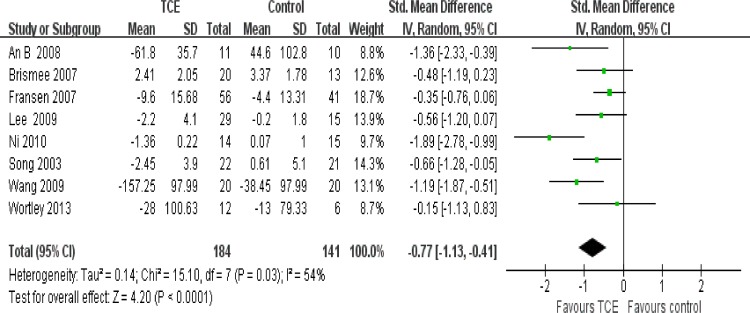
Forest plot comparison: 1 pain, short-term, outcome: 1.1 TCE versus control.

**Fig 5:**
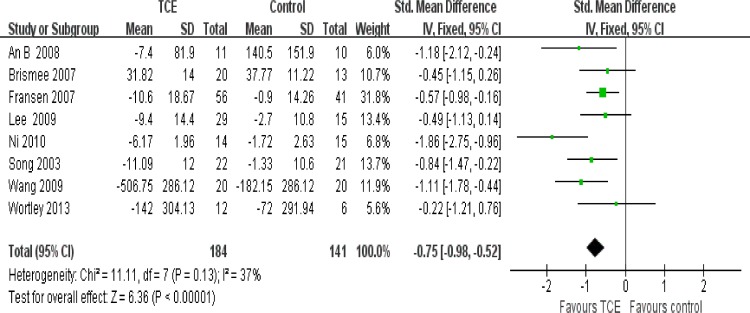
Forest plot comparison: 2 physical function, short-term, outcome: 2.1 TCE versus control.

**Fig 6:**
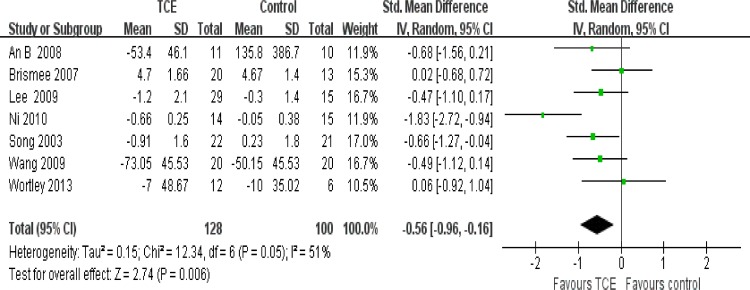
Forest plot comparison: 3 stiffness, short-term, outcome: 3.1 TCE versus control.

**Fig 7:**
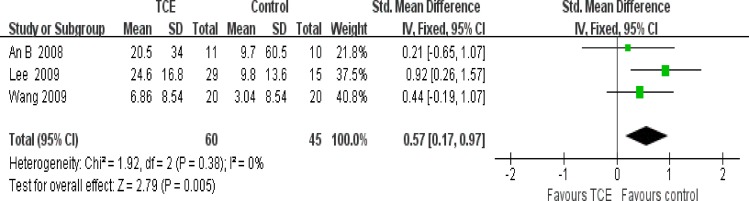
Forest plot comparison: 4 quality of life, short-term, outcome: 4.1 TCE versus control.

**Fig 8:**
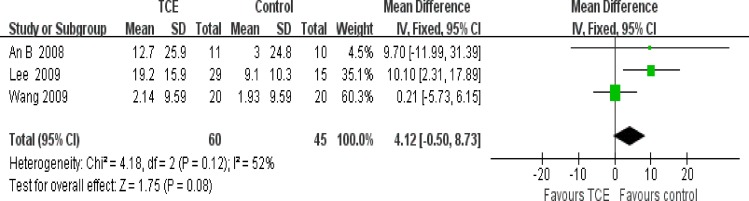
Forest plot comparison: 5 mental health, short-term, outcome: 5.1 TCE versus control.

The effect of short-term TCE is shown in Figs 4–8. Pooling of the data for short-term TCE using a fixed effect model revealed a moderate effect size for pain relief (SMD: -0.77;95% CI: -1.13 to -0.41; P<0.0001), physical function improvement (SMD -0.75; 95% CI: -0.98 to -0.52; P<0.00001), stiffness alleviation (SMD: -0.56; 95%: CI -0.96 to -0.16; P<0.006) and no significant effect size for quality of life improvement (SMD: 0.57; 95% CI: 0.17 to 0.97; P = 0.005), and mental health improvement (SMD 4.12; 95% CI: -0.50 to 8.73; P = 0.08). The study heterogeneity was calculated as I^2^ = 54%, 37%, 51%, 0%, and 52% for pain, physical function, stiffness, quality of life, and mental health, respectively. There was not enough evidence to prove any long-term effects of TCE on knee OA. In addition, our results revealed that TCE was not associated with serious adverse events.

### Sensitivity analyses

After sensitivity analysis, we found that except the outcomes of quality of life and mental health, the rest of the meta-analysis did not change the results, suggesting that the outcomes of quality of life and mental health were not stable, and the results were needed to be carefully interpreted. Besides, We also found that the statistical heterogeneity of pain, physical function and stiffness were most probably caused by the study of Ni [52], and the study of Lee [51] and Wang [57] had led to the statistical heterogeneity of the quality of life and mental health, therefore special attention should be paid to them in the analysis of the results.

## Discussion

### Summary of evidence

Our meta-analysis of the current literature showed that short-term TCE was potentially beneficial in terms of reducing pain, improving physical function, alleviating stiffness. No long-term effects in the relevant studies were observed. Available safety data suggested that short-term TCE was not associated with serious adverse events. Thus, the safety and efficacy of short-term TCE suggest that it might be useful as an adjuvant treatment for patients with knee OA.

Other systematic reviews that have been conducted on TCE have focused more on specific types of exercises such as Tai Chi [33,34]. In contrast, this review was based on a number of trials aimed to determine the effectiveness of TCE for the treatment of knee OA so as to explore an alternative and less strenuous treatment for patients with knee OA. The results of this review support Lauche’s review, which reported improvements in pain, physical function and stiffness with short-term Tai Chi [33]. Further, our review was also consistent with Kang’s review, which reported the effectiveness of Tai Chi in controlling pain and improving physical function [34]. This may largely be due to previous studies only exploring Tai Chi, as well as to the general lack of high quality literature. Further, Kang’s review did not cover the effects of long-term Tai Chi exercise. Although Lauche’s review improved on Kang’s study, in that it included reports that evaluated the effects of long-term Tai Chi, the review did not explore the effects of other TCEs.

It should be noted that, of the articles included in this review, there was considerable variation in the duration of TCE training programs, with interventions ranging from 8 to 24 weeks in length. Here, we determined that 12-week interventions were most common and that no significant effects existed between training sessions of 12 weeks and those lasting longer than 12 weeks in their efficacy of relieving pain, improving physical function and alleviating stiffness in patients with knee OA. In terms of TCE frequency, programs employing TCE twice a week were most common in the studies we reviewed. This moderate weekly frequency could contribute to the observed increase in muscle hypertrophy and power gains in older adults [64].

The underlying mechanisms of TCE in treating knee OA are complex and based on the theoretical principles of traditional Chinese medicine. Such principles focus on integrating exercise of the mind and body, which includes improving breath flow, calming the mind, and self-correcting posture and body movements, in order to activate the body’s ability to self-heal and to evoke a balanced release of endogenous neurohormones and various kinds of natural health recovery mechanisms [65]. TCE is not only effective for the knee, but through the the exercise of whole body to improve the overall function. Nevertheless, the contribution of TCEs to the health of patients with knee OA needs further investigation.

In terms of the pooled estimates of pain, stiffness, quality of life and mental health, no significant heterogeneity was found. However, substantial heterogeneity was found in pain and stiffness, moderate heterogeneity was found in physical function. In this regard, we had conducted a series of sensitivity analyses and found that the heterogeneity was obviously decreased after excluding the study of Ni [52]. After comparison of the characteristics of these studies included, we speculated that there were mainly the following reasons: above all, we observed major differences in duration and frequency.The duration of Ni’s study [52] was two times as long as 24 weeks of the other studies. Further, different types of TCE have specific forms and functions. For example, Tai Chi can be divided into Yang-style and Chen-style. In addition, participants from different countries and most of the researches mainly concentrated in the United States which may have had diverse understandings of TCE. And it would lead to very different ways of exercise even for the same kind of traditional exercises. Besides, most of the sample size of the studies included were small. Finally, some of the observed differences were likely due to important clinical and methodological variations. These were perhaps the cause of the instability of the outcomes of the quality of life and mental health. What’s more, we speculated that the lack of relevant researches and the small sample size may be the biggest reason. In the two outcomes, Lee’s [51] and Wang’s [57] study had led to the larger heterogeneity respectively. However, in terms of the small number of studies, and the significance of the two outcomes may need a long time to observe due to the TCEs may play an indirect role in improving the quality of life and mental health by regulating the whole body function, We considered there was little significance on the results of these two outcomes.

Of the studies included in this review, lack of double blinding was the most common source of methodological bias. In fact, three studies failed to indicate allocation concealment in their experimental procedures suggesting the possibility of bias in their selection process. Such bias could have led to a placebo effect for patients with knee OA, which for patients who known that they had received treatment and the subjectively felt effective or the same for the doctors, reducing credibility of the study results. It should be noted that excluding studies published in languages other than Chinese or English likely caused us to miss relevant studies, which may have influenced our results.

### Limitations

Our study was potentially limited severely by the lack of the large number of reviewed TCE types, as well as any significant long-term effects of TCE. Besides, all of the 8 cases included in this study are small sample research, and lack of large sample RCT research basis that will inevitably affect the level of evidence, especially for the instability of the outcomes of the quality of life and mental health. Therefore, a large number of high quality relatively studies is needed to clarify this issue.

## Conclusions

This study was probably the first systematic review and meta-analysis to determine the effects of TCE for the treatment of knee OA. Although it has certain limitations, the study might have proved that short-term TCE can reduce pain, improve physical function and alleviate stiffness. Further, this review has demonstrated that there might be no doubt in the effectiveness and safety of TCE, suggesting that it was potentially useful as an adjuvant treatment for patients with knee OA. More studies are urgently needed to confirm these results, to determine whether the positive effects of TCE can be supported by appropriately designed studies with long-term follow-ups.

## Supporting Information

S1 File The full electronic search strategy. (DOC)

S2 File PRISMA checklist. (DOC)
