# Supplementary material for: The Effects of Traditional Chinese Exercise in Treating Knee Osteoarthritis: A Systematic Review and Meta-Analysis
Source: PLoS One. 2017 Jan 25;12(1):e0170237. doi: 10.1371/journal.pone.0170237 (PMC5266306; doi:10.1371/journal.pone.0170237)
Supplement: S1 File — (DOC) [file pone.0170237.s001.doc]

**The full electronic search strategy**

**1.PUBMED**

("osteoarthritis, knee"[MeSH Terms] OR ("osteoarthritis"[All Fields] AND "knee"[All Fields]) OR "knee osteoarthritis"[All Fields] OR ("osteoarthritis"[All Fields] AND "knee"[All Fields]) OR "osteoarthritis, knee"[All Fields]) AND ("TCE"[All Fields] OR "baduanjin"[All Fields] OR ("yi"[All Fields] AND "jinjing"[All Fields]) OR "wuqinxi"[All Fields] OR "Tai Chi"[All Fields] OR "T'ai-chi"[All Fields] OR "Tai Ji"[All Fields] OR "Taiji"[All Fields] OR "Taiji quan"[All Fields])

1. **EMBASE**

#1 knee osteoarthritis

#2 traditional exercise

#3 baduanjin

#4 yijinjing

#5 Taiji

#6 Tai Chi

#7 T’ai-chi

#8 Tai Ji

#9 Taiji quan

#10 #2 OR #3 OR #4 OR #5 OR #6 OR #7 OR #8 OR #9

#11 #1 AND #10

1. **THE COCHRANE CENTRAL REGISTER OF CONTROLLED TRIALS**

#1 knee osteoarthritis

#2 (traditional exercise or baduanjin or yi jinjing or wuqinxi or Taiji or Taichi or T’ai-chi or Tai Ji or Taiji quan)

#3 #1 and #2

1. **CHINESE BIOMEDICAL LITERATURE DATABASE**

**#1 膝骨关节炎**

**#2 传统运动疗法 OR 八段锦 OR 易筋经 OR 五禽戏 OR 太极**

**#3 （#1）AND（#2）**

1. **THE WEB OF SCIENCE**

(Osteoarthritis, Knee)AND(traditional exercise OR baduanjin OR yi jinjing OR wuqinxi OR Tai Chi OR T'ai-chi OR Tai Ji OR Taiji OR Taiji quan)
